# Expert perspectives on essential parameters to monitor during childbirth in low resource settings: a Delphi study in sub-Saharan Africa

**DOI:** 10.1186/s12978-019-0786-6

**Published:** 2019-08-05

**Authors:** Michael S. Balikuddembe, Nazarius M. Tumwesigye, Peter K. Wakholi, Thorkild Tylleskär

**Affiliations:** 1 Centre for International Health, University of Bergen, P O Box 7800, 5020 Bergen, Norway; 2 Department of Obstetrics and Gynaecology, Mulago National Referral and Teaching Hospital, P O Box 7051, Kampala, Uganda; 3 Department of Epidemiology and Biostatistics, Makerere University School of Public Health, P O Box 7072, Kampala, Uganda; 4 College of Computing and Information Science, Makerere University Kampala, P O Box 7062, Kampala, Uganda

**Keywords:** Childbirth monitoring, Partograph, Expert opinions, Delphi study, Consensus, Suivi de l’accouchement, Partogramme, Avis d’experts, Étude Delphi, Consensus

## Abstract

**Objective:**

There is no consensus on the essential parameters to monitor during childbirth, when to start, and the rate of monitoring them. User disagreement contributes to inconsistent use of the twelve-item modified World Health Organization partograph that is started when the cervix is at least 4 cm dilated. The inconsistent use is associated with poor outcomes at birth. Our objective was to identify the perspectives of childbirth experts on what and when to routinely monitor during childbirth in low resource settings as we develop a more acceptable childbirth clinical decision support tool.

**Method:**

We carried out a Delphi study with two survey rounds in early 2018. The online questionnaire covered the partograph items like foetal heart, cervical dilation, and blood pressure, and their monitoring rates. We invited panellists with experience of childbirth care in sub-Saharan Africa. Consensus was pre-set at 70% panellists rating a parameter and we gathered some qualitative reasons for choices.

**Results:**

We analysed responses of 76 experts from 13 countries. There was consensus on six important parameters including foetal heart rate, amniotic fluid clearness, cervical dilation, strength of uterine contractions, maternal pulse, and blood pressure. Two in three experts expressed support for changing the monitoring intervals for some parameters in the partograph. 63% experts would raise the partograph starting point while 58% would remove some items from it. Consensus was reached on monitoring the cervical dilation at 4-hourly intervals and there was agreement on monitoring the foetal heart rate one-hourly. However, other parameters only showed majority intervals and without reaching agreement scores. The suggested intervals were two-hourly for strength of uterine contractions, and four-hourly for amniotic fluid thickness, maternal pulse and blood pressure. The commonest reason for their opinions was the more demanding working conditions.

**Conclusion:**

There was agreement on six partograph items being essential for routine monitoring at birth, but the frequency of monitoring could be changed. To increase acceptability, revisions to birth monitoring guidelines have to be made in consideration of opinions and working conditions of several childbirth experts in low resource settings.

**Electronic supplementary material:**

The online version of this article (10.1186/s12978-019-0786-6) contains supplementary material, which is available to authorized users.

## Abstrait

**Objectif:**

Il n’ya pas de consensus sur les paramètres essentiels à surveiller pendant l’accouchement, quand commencer et le taux de surveillance. Le désaccord des utilisateurs contribue à une utilisation incohérente du partogramme modifié de l’Organisation mondiale de la Santé en douze éléments qui est. démarré lorsque le col. de l’utérus est. dilaté à au moins 4 cm. L’utilisation incohérente est. associée à de mauvais résultats à la naissance. Notre objectif était d’identifier les points de vue des experts en matière d’accouchement sur ce qu’il convient de surveiller régulièrement pendant l’accouchement dans les pays à faibles ressources, et à quel moment, afin de développer un outil d’aide à la décision clinique plus acceptable pour l’accouchement.

**Méthode:**

Nous avons mené une étude Delphi avec deux enquêtes au début de 2018. Le questionnaire en ligne couvrait les éléments du partogramme tels que le cœur du fœtus, la dilatation du col. utérin et la pression artérielle, ainsi que leurs taux de surveillance. Nous avons invité des intervenants expérimentés dans les soins à l’accouchement en Afrique subsaharienne. Le consensus était prédéfini à 70%. Les membres du panel évaluaient un paramètre et quelques raisons qualitatives des choix recueillis.

**Résultats:**

Nous avons analysé les réponses de 76 experts de 13 pays. Un consensus s’est. dégagé sur six paramètres importants, notamment la fréquence cardiaque fœtale, la clarté du liquide amniotique, la dilatation du col. utérin, la force des contractions utérines, le pouls maternel et la pression artérielle. Deux experts sur trois se sont déclarés favorables à la modification des intervalles de surveillance pour certains paramètres dans le partogramme. 63% des experts élèveraient le point de départ du partogramme alors que 58% en supprimeraient certains éléments. Un consensus s’est. dégagé sur le suivi de la dilatation cervicale toutes les 4 heures et un accord sur le suivi du rythme cardiaque fœtal toutes les heures. Cependant, d’autres paramètres ne montraient que les intervalles de majorité et sans atteindre les scores d’accord. Les intervalles suggérés étaient de deux heures pour la force des contractions utérines et de quatre heures pour l’épaisseur du liquide amniotique, le pouls de la mère et la pression artérielle. La raison la plus commune de leurs opinions était les conditions de travail les plus exigeantes.

**Conclusion:**

il y avait accord sur le fait que six items du partogramme étaient essentiels pour le suivi systématique à la naissance, mais la fréquence du suivi pourrait être modifiée. Pour accroître l’acceptabilité, il est. nécessaire de réviser les lignes directrices sur la surveillance des naissances en tenant compte des opinions et des conditions de travail de plusieurs experts en matière d’accouchement dans des pays à faibles ressources.

## Plain English summary

There is disagreement on the essential items to monitor during childbirth and when to monitor them, which results in unwanted birth outcomes. The World Health Organisation recommends that regular monitoring of 12 or so items during labour should start when the opening of the cervix reaches four centimetres, and continue at intervals of 30 min to four hours. We set out to identify the opinions of childbirth experts on the recommendations.

We carried out a two-round online survey in early 2018. We asked for opinions about items like foetal heart sounds, opening of the uterine cervix, the mother’s blood pressure, and the frequency of monitoring them. Participants were childbirth experts who had worked in sub-Saharan Africa for at least one year.

Seventy-six experts from 13 countries completed the first round of the survey while 16 completed the second round. The agreed upon important items were foetal heart sounds, opening of the uterine cervix, clearness of the water around the baby, strength of uterine contractions, maternal pulse, and blood pressure. Two in three experts did not agree with the recommended monitoring intervals. For example, most of them would rather monitor the foetal heart sounds every one hour instead of every half hour, and monitor the other important items after every four hours. The commonest reason for their opinions was the more demanding working conditions.

There was agreement on six of twelve items as being essential for routine monitoring at birth, but the frequency of monitoring could be changed. Revisions to birth monitoring guidelines have to be made with consideration of opinions and working environments of childbirth specialists.

## Introduction

About 303,000 women and a higher number of babies died in 2015 from pregnancy-related causes [1] like obstructed labour. Obstructed labour directly contributes 6–8% maternal deaths but it plays a role in other causes of death and morbidity for mother and baby [2, 3]. Over 95% of this morbidity and mortality occurs in low- and middle-income countries (LMIC), with over 35% found in East and Southern Africa [1, 4].

At least 80% of the poor pregnancy outcomes are preventable through interventions like adequate monitoring of the labour and delivery process [2, 5, 6]. In sub-Saharan Africa (SSA), the labour monitoring is often inadequate as evidenced by poor documentation and outcomes of labour [4, 7, 8]. The monitoring is hampered by lack of user-friendly tools for labour management, limited access to evidence-based clinical guidelines for the providers and users of maternal health services, maternity provider factors, weak referral networks and limited health financing [9, 10]. The partograph has been promoted by the World Health Organisation (WHO) as the standard labour monitoring tool [6] but its use is still poor due to many user challenges [8, 11, 12]. It was designed to be an easy-to-use aid for use by expert and non-expert birth attendants across maternity service delivery points [6]. It is a paper tool with over 12 parameters for monitoring labour progress, foetal condition and maternal status at intervals of 5 min to 4 h [13, 14]. The parameters are often based on weak evidence that there are no studies to support the starting point and optimal frequency of examinations for the foetal heart and cervical dilation which are the most measured parameters [14–17].

To address usability, some researchers have suggested simpler childbirth monitoring without adverse effects on pregnancy outcomes [5, 15, 18]. Moreover, other authorities called for a revamp of the partograph citing changed physiology of labour over time [19, 20], which attracted a backlash from traditionalists and realists [21–23]. Other researchers recommended the cessation of using community-generated childbirth monitoring curves in making decisions for individuals [24]. A review of the computerized childbirth monitoring tools found a limited number of them but they were not suited to the diverse birth monitoring contexts in SSA [25]. The WHO called for research into other paper or digital labour monitoring tools that are more efficacious and acceptable to maternity service providers to guide clinical decisions, avoid excessive interventions and improve birth outcomes [20, 26]. This research was part of a project to develop and evaluate a mobile tool (electronic or otherwise) to assist in childbirth monitoring. In view of the lack of consensus on the parameters to include for monitoring we decided to conduct this study. In this study, our aim was to identify the agreeable essential items to monitor during normally progressing childbirth, and the acceptable frequencies of monitoring them, for inclusion in a childbirth monitoring decision support tool.

## Methods

### Design and study setting

We used an online modified Delphi technique with two survey rounds. A classic Delphi survey has an initial exploratory round for identifying debatable issues and one or more iterative question-answer rounds for experts to determine the level of support or to approach a consensus [27–29]. We did not have the exploratory round since the partograph issues were widely published. Besides, we didn’t seek new parameters for childbirth monitoring but rather the identification of the (non)contentious ones and seek convergence. The initial round was informed by a synthesis of the existing childbirth management guidelines and literature review on partograph use [2, 7, 15, 18, 19, 21, 23, 25, 30]. A synopsis was provided to potential participants in the invitation email and in the introduction of the questionnaires. To achieve a good response rate and lower the dropout rate, classic Delphi studies need a lot of time during and between rounds [27]. The study duration can be markedly reduced through modifications like digitization [28], hence we chose to use the online Delphi method.

### Study participants

The survey respondents were experts in childbirth care. The qualities of an expert vary with subject matter. For this study, a respondent had to have at least 12-months experience of maternity service in low-income settings of sub-Saharan Africa. Another criterion was the ability to understand and communicate in English. We emailed invitations for participating in the survey to doctors and midwifery care providers directly or through their professional organizations and to selected authors in maternal health publications.

Purposive sampling was used to identify potential expert participants from websites of Obstetrics and Gynaecology societies. We sent / routed invitations to/through persons listed on various country or international society websites as secretaries or presidents of obstetrics and gynaecology societies in SSA. In round 1 we sent 213 direct invitations and an unknown number through 4 professional associations, while for the second round we sent direct invitations to experts who had expressed willingness to participate in round 2. Although the minimum required number of participants was 15 respondents for a survey round, we invited a much higher number to increase inclusivity as well as offset the known low response and high attrition rates during Delphi studies [31].

We collected data on demographic characteristics of participants such as one’s professional training, and length maternity service cum experience in labour monitoring. Furthermore, we gathered the suggestions on parameters to monitor at childbirth and reasons given to support them.

### Data collection and analysis

**Fig. 1:**
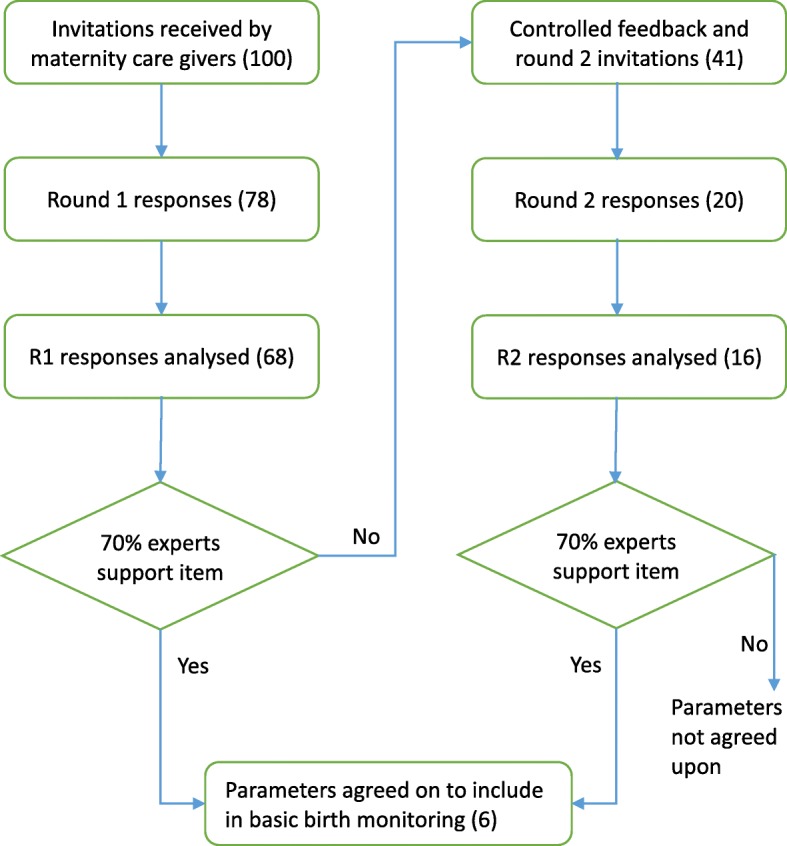
Flow diagram from expert invitation to proposed essential childbirth monitoring parameters in sub-Saharan Africa

Semi-structured questionnaires, see Additional files 1 and 2, (with both limited and unrestricted answer options) based on the modified WHO partograph and labour monitoring guidelines in the integrated management of pregnancy and childbirth guide were used [30]. It contained two main sections addressing the importance of parameters and the frequency of measuring each parameter. The scale for rating the importance of a parameter had five points from “not important” to “maybe important” to “slightly important” to “moderately important” to “very important”. For the rate of assessments during childbirth monitoring, the panellists were presented a six-point scale, from every 30 min to over 4 h, against which to rate each item. The questionnaire was pretested among maternity providers, including a midwife, a medical officer and an obstetrician, who were ineligible to participate in the survey. The experts had 4 weeks in which to respond or change responses. Reminders were sent out to those who hadn’t completed the survey round at 2 weeks, 1 week and 2 days from closure of a survey round. In round one, consensus was set a priori at 70% or more of panellists scoring a parameter in the highest point of the Likert-type scale [28]. For the second round responses, consensus was set at a score of 70% or more within two Likert scale points and some qualitative explanations for the numerical responses were assessed. After analysing the first round responses, the panellists were sent results and requested to reconsider questions where there was no consensus yet (Fig. 1).

## Results

### Round 1 results

At least 100 invitations reached the target providers but we got 76 eligible respondents from 13 countries with a questionnaire completion rate of 89%. At least three of those who completed the survey were midwives. The median age was between 35 and 44 years while the mean duration of maternity service in SSA was 10–15 years with a median between 6 and 10 years. Most panellists worked in referral and teaching hospitals with inadequate maternity staff numbers. Four in five panellists attended to at least one birth in a week. Additional demographics of the respondents are shown in Table 1.

**Table 1:** Demographic characteristics of the respondents

Characteristic	Number of experts	Percentage
Age, *n* = 68		
25–34	17	25.0
35–44	24	35.3
45–54	22	32.4
55–64	5	7.4
Years of maternity care in sub-Saharan Africa, *n* = 68		
1–5	9	13.2
6–10	26	38.2
11–20	20	29.4
Over 20	13	19.1
Country of professional society		
Uganda	29	42.6
Kenya	19	27.9
Rwanda	5	7.4
Botswana	4	5.9
Mozambique	3	4.4
Tanzania	3	4.4
Others	9	13.2
Place of work, *n* = 68		
Teaching hospital	38	55.9
Referral hospital	32	47.1
Private for profit	16	23.5
Private not for profit	8	11.8
Public facility	25	36.7
Urban facility	11	16.2
Health centre / unit	2	2.9
Has enough staff to monitor labour	5	7.4
Frequency of managing labour, *n* = 65		
At least 1 per week	53	81.5
At least 1 per month	8	12.3
At least 1 in 3 months	4	6.2

**Fig. 2:**
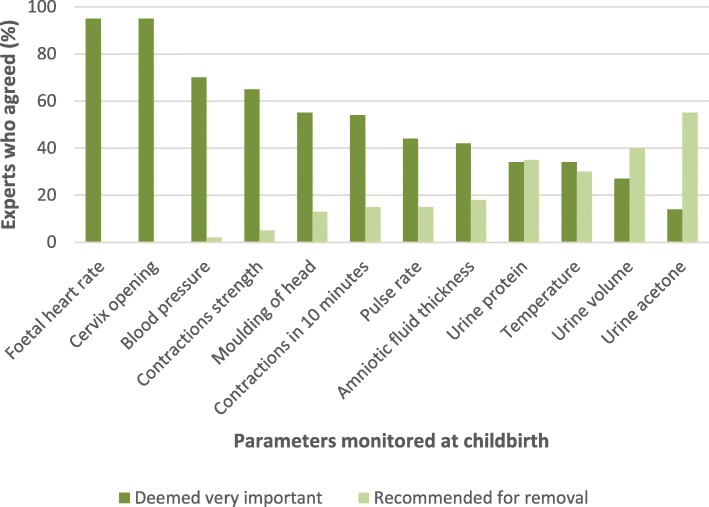
Parameters deemed very important and those recommended for removal from childbirth monitoring in round one

The questions on parameter importance were answered by 65 respondents, while 60 experts answered the questions on reducing number of items monitored routinely. Foetal heart rate (FHR), cervical dilation, and maternal blood pressure (BP), reached the consensus score (70%) in the first round as very important parameters to monitor at birth. Asked to suggest parameters for removal from routine monitoring, most experts chose urine acetone and urine volume. This information is presented in Fig. 2. However, 2 in 5 experts would maintain all items on the partograph. Although not requested, two experts suggested new items to be included in the tool. The items are bladder state (full or empty), position or malposition of foetal head, and the examiner’s initials below the time of plotting.

Sixty-four experts answered the questions on interval of monitoring items where 66% supported and 12% were undecided on the idea of changing the rates of monitoring different items. There was no consensus on monitoring intervals as shown in Table 2. Most panellists elected to monitor cervical dilation at 4-hourly intervals, 30 min for foetal heart rate, and 4 or more hourly for urine parameters, amniotic fluid, moulding, and foetal descent. There was no clear pattern for maternal pulse, contractions, and temperature.

**Table 2:** Suggested monitoring interval for each parameter and the proportion of experts who agreed with it

Parameter	Monitoring interval (Hours)	Experts in agreement (%)
Foetal heart rate	0.5	65
Amniotic fluid	4	56
Moulding of head	4	53
Cervix opening	4	67
Contractions in 10 min	0.5	40
Contractions strength	0.5	40
Descent of foetus	4	47
Pulse rate	0.5	35
Blood pressure	4	52
Temperature	2	40
Urine volume	4	39
Urine acetone	Over 4	56
Urine protein	Over 4	65

Reasons given for the expert opinions were of two main categories, namely; unrealistically high monitoring rates for the workforce, and unproven benefit of some parameters, divergent patterns of labour.

“*The frequency of monitoring most of the parameters for maternal well-being is more than what is necessary for sensitivity in our setting and thus not aligned to the practical realities of medical practice.”* (Feb 09, 02:21 AM)

*“Our health unit settings are completely different from what the WHO partogram is meant for. The motivation of health workers to monitor on a partogram is also very low. In a health centre II or III the midwife cannot practically sit down and monitor FHR every 30 minutes.”* (Feb 15, 02:02 AM)

Subgroup analysis of the data showed no significant difference in results when responses of experts with 1–5 years’ experience were omitted. However, the importance score of BP did not make the cut-off of 70% when the junior experts were removed. Contractions in 10 min and temperature were also rated less important by the more experienced group. Of the 12 experts who performed one or less births per month, 75% agreed with a suggestion to change the monitoring frequencies for various parameters. Four would monitor FHR every 30 min, two suggested one-hourly intervals, another three preferred 2-hourly intervals. They preferred monitoring contractions at interval of 3 or more hours. The other parameters were similar to that of the average participant. After this analysis, the parameters below the consensus score were presented to the experts for reconsideration of their importance and rates of monitoring. 41 experts expressed their willingness to be participate in the second round of the survey.

### Round 2 results

All 41 invited experts received the round 2 questionnaire but 19 responded and the completion rate was 84%. The 16 experts whose responses made the analysis were from 8 countries. The respondent characteristics were similar to the first round group. The average duration of maternity service in SSA was 9 years with a median within the 6–10-year bracket.

**Fig. 3:**
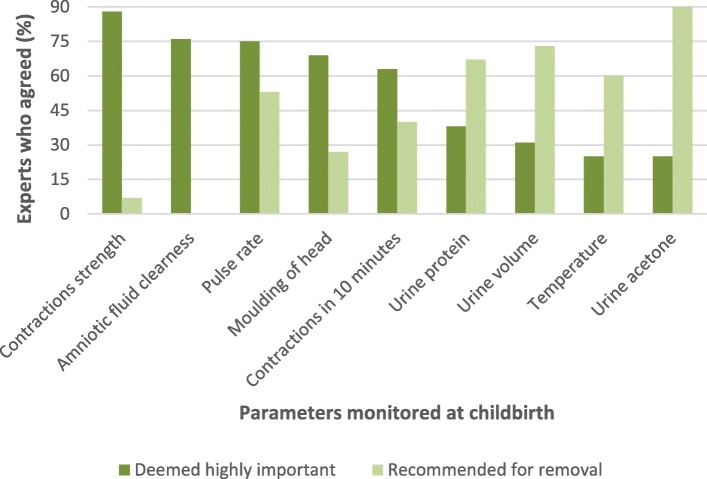
Highly important parameters and those recommended for removal from routine childbirth monitoring in round two

Figure 3 indicates the proportions of respondents who felt that some parameters were important for routine childbirth monitoring in round two. It also shows the trend of what they felt could be removed from regular monitoring. The parameters agreed upon in round one were not presented for consideration and are omitted from this figure.

**Fig. 4:**
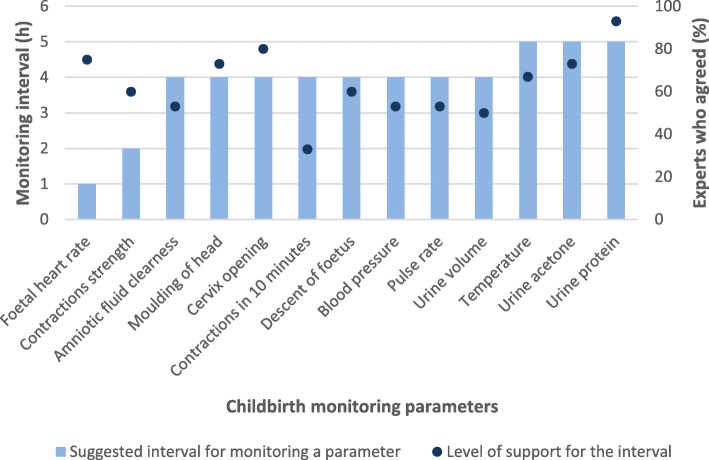
Monitoring intervals for parameters and the percentage of round 2 experts in agreement

Asked about a need to change the monitoring intervals for the parameters, 93% experts responded in the affirmative. The intervals suggested by most experts for each parameter and the proportion of respondents who agreed with the interval are also depicted in Fig. 4. There was agreement on the monitoring intervals for FHR (1 h, 75%), moulding of the skull (4 h, 73%), cervical dilation (4 h, 80%), urine acetone (over 4 h, 73%) and urine protein (over 4 h, 93%). The majority of the respondents expressed support for removing the temperature (60%), urine protein (67%), volume (73%), and acetone (93%) from routine monitoring at birth. Sixty-seven percent of the experts agreed with calls to raise the starting cervical dilation for active labour while 53% encouraged the use of general alert and action lines to make clinical decisions for individual women.

Generally, the expert opinions did not change much between rounds. Even the further consideration and adjustment of results in round 2, the majority opinions were unaffected. Only three parameters were added to the essentials list. For the monitoring frequencies, foetal heart rate was the only important parameter to have a significant change, that is, from half-hourly to one-hourly.

## Discussion

Over the 2 rounds, the panellists elected to monitor cervical dilation (4-hourly), strength of uterine contractions (2-hourly), foetal heart rate (1-hourly), thickness of amniotic fluid (4-hourly), maternal pulse (4-hourly), and BP (4-hourly). Considering the WHO recommendations in the modified partograph [30], a significant reduction in the monitoring frequency was noted for foetal heart rate, maternal pulse, and uterine contractions. Recently, the WHO reiterated the guidelines for FHR monitoring as every 15–30 min during first stage of labour which contrasts our finding of 60-min intervals [20]. A study similar to ours was conducted around the same period as we did and found consensus on monitoring foetal heart every 30 min in low risk active labour [32]. It was a Delphi study that focused on foetal heart monitoring (FHM) in low income countries, but less than 10% of participants were from low income countries. Moreover, 12% participants lacked experience in low resource settings and another 10% had less than 1 year of experience in those settings. In a study by the same authors, where most participants were from a low resource setting, the agreed upon FHM interval was one-hourly like in our study [33]. Therefore, the differences in FHM intervals could be due to the settings of origin for most participants in the consensus process whereby those from low income settings favour the higher intervals and vice versa. The 4-h interval for monitoring cervical dilation was the same as that agreed upon by the WHO Guideline Development Group although it also lacked direct evidence to support its recommended interval, the group stressed the need for minimising vaginal examinations during labour [20].

Partograph completion studies variably indicate that cervical dilation, contractions, foetal descent, and foetal heart are the most recorded and perhaps monitored parameters, which partly agrees with our findings on preferred parameters [4, 7].

Round one generated three high scoring parameters but no definitive monitoring frequencies. This suggests that the three, namely cervical dilation, FHR and BP, are undoubtedly the most essential parameters to the experts. We used round 2 to give feedback, allow expert reflections and allow room for other opinions especially on the rates of monitoring. This practice is good for consensus generation on divisive subjects particularly among subjective issues like determinants of childbirth outcomes [27, 31]. Round 2 turned out to be a confirmation of the opinions from round 1, except for the temperature, amniotic fluid, and moulding of the skull. Therefore, the low response rate in round 2 may not have affected the agreed upon childbirth monitoring parameters. This aligns with researchers who advise that, although the larger the better, a Delphi panel size above four is adequate, if the panellists have comparable knowledge on the subject [28, 31]. Moulding missed the cut off for agreement by one point hence it was difficult to make a substantial conclusion about its usefulness. Significant moulding is associated with obstructed labour and perinatal morbidity or mortality hence it should be assessed for at every internal pelvic examination [30].

As the phenomenon of childbirth is better understood and in the face of diverse settings of labour, the debate on the necessary monitoring during normal childbirth to prevent poor outcomes is unavoidable [34]. Even within comparable contexts like SSA, there is agreement on some parameters to monitor and new research seeks to answer the unresolved issues [24]. As such, the current WHO partograph may not be suitable for assessing the quality of childbirth monitoring. The disagreements on what constitutes essential childbirth monitoring led experts in our study to support the WHO appeals for research on the ideal tool for labour monitoring to guide decision making [20], and the calls for individualised childbirth monitoring [24]. Many expert opinions hinged on experience from working in low resource conditions and inadequate evidence to support present recommendations. This was similar to findings of other studies [29] and implied that the suggestions can still change as additional resources and evidence for practice are realized.

The most contested parameter is the FHR monitoring interval with a thin line between agreement and disagreement on the 30- versus 60-min intervals which was also evident in our findings. The key question is whether the sixty-minute monitoring interval would not increase poor foetal outcomes compared to the 15- or 30-min intervals during the active phase of first stage of labour. From some clinical observations and prospective studies it was shown that a 60-min interval may not be bad for the foetus (with a normal placenta) but may be safer for the mother than the shorter intervals [3, 17, 35–38]. In a national survey it was found that there was no difference in clinical outcomes for diagnosis to delivery interval of 16–75 min in women receiving Caesarean section which were mostly due to foetal distress [37]. In Uganda, a survival analysis was done for babies born through emergency C-section and results indicated that foetal outcomes did not differ within 2 h of a decision for emergency C-section [3]. It was also shown that a normal foetus with a normal placenta is able to withstand heart beat drops of 15 beats for 1 min up to 72–84 times within the 2 h preceding delivery [36]. In reality, the sudden severe bradycardia and prolonged decelerations are very rare and follow acute events, like placenta separation, cord compression after rupture of membranes, and uterine rupture, which are easily picked. Therefore, the one-hour FHR monitoring interval agreed upon by the experts in our survey will not necessarily lead to poor newborn outcomes. Further discussion is also needed on the significance of monitoring foetal skull moulding. For the time being, we may have to use consensus-based guidelines as we research for the better data based ones.

Taking one step back and looking at the big picture, it is obvious that in better-resource settings there is a continuous drive towards more labour monitoring as part of defensive medicine against litigation [39]. The question is how far from “maximum monitoring” a decision support tool can be. This is particularly true for the monitoring intervals the tool suggests. Most likely, it must be possible to adjust the monitoring intervals to the local circumstances in each childbirth unit. Regarding the parameters to monitor, it may be that some appear up front on the tool and others – considered less important – appear in a more hidden place.

In this study, we had strengths and limitations. The main strengths of this study were the incognito exchange of opinions and the inclusion of experts from countries in the same region. Confidentiality of respondents was a key consideration since in clinical care the opinions of junior staff are sometimes suppressed by the seniors who may not have up to date evidence for decisions. Unlike global online studies [29, 32], our respondents were from the same geographical and socioeconomic region to ensure as similar working conditions as possible to give a more realistic opinion.

The first limitation was the low response rate in round two. This could have reduced our process gain since long term consensus is achieved through high numbers of participants. Although the questionnaire completion rate was good, considering the importance of the study subject, we received fewer than anticipated respondents, even though we were within the model panel size for Delphi studies [27–29]. This could have been due to inability to access internet connections but also residual normative and informational pressures that prevent experts from participating unreservedly. A confident respondent may have answered as an expert yet confidence is a signal of status rather than a valid indicator of expertise. More so, a less confident or strategically “static” expert may have held back valid information or a minority opinion that could have swayed the final outcome towards the truth. Due to variation in the completion rates across questions, our unit of data analysis was the question in order to include as many expert opinions as possible. Hasson et al. (2000), state that the response rates may be increased by pursuing non-responders via reminders [27]. However, this may be counterproductive to anonymity and it could increase normative pressures towards consensus, hence we limited it to avoid an impression of soliciting expert opinions [28] and chose to extend the survey duration. Some researchers conduct consensus meetings to try and mitigate low response rates for Delphi studies like we faced [32]. A consensus meeting is useful if there is persistent non-consensus or a conflict between the majority opinion on the best medical practice and ethical concerns about this practice. The researchers’ biases are reduced through critical reflection on outcomes within the team and having a final draft of the outcomes reviewed by an external board or authority before publication and dissemination [40]. We reflected on the results but unfortunately the global authority on such matters (the WHO) has conceded that more research is needed on the best maternity practice which was also a justification for this study. The second limitation was non-separation of the survey questions on monitoring frequencies for the first and second stages of labour. Though it is a much shorter part of normally progressing labour, the second stage is equally important and the monitoring frequencies may differ from those in the first. Being part of the secondary objective, we left it for panellists to determine in the “other frequency” option, but only two commented about second stage moreover they declined further participation. Another reader may consider our non-classification of the recommendations for high and low risk labours as a limitation. Labour can only be classified as low risk (normal) after it is complete. The guidelines in the WHO partograph are intended for the mothers/foetuses expected to go through labour without distress. Once a mother or foetus gets distressed, the necessary interventions have to be made according to the identified risk(s). For this reason, there is no and it is unlikely to gather consensus on monitoring intervals for the higher risk labours [14, 16, 17, 20, 32, 41]. Another study limitation was the low number of midwives who participated. It could have been due to our failure to send direct invitations to more midwives or their professional societies.

## Conclusions

According to the childbirth experts in this study, the essential items to monitor during normally progressing childbirth were cervical dilatation, strength of uterine contractions, foetal heart rate, amniotic fluid thickness, maternal pulse rate, and blood pressure. These items and the proposed monitoring intervals vary from the standards in the modified WHO partograph but they are similar to childbirth monitoring guidelines used in some other resource limited settings. Although more research is needed on the study subject, with roots in low resource maternity units, these guidelines could be more practical, achievable and enforceable in low income settings than the current WHO and international guidelines. As we await new evidence, it is worthwhile including expert perspectives in the mobile child birth monitoring tools for use in maternity centres with skilled staff constraints.

## Additional files

Additional file 1: Round 1 Questionnaire. (XLSX 32 kb)

Additional file 2: Round 2 Questionnaire. (XLSX 23 kb)

## Data Availability

The anonymized data sets are available in the supplementary material section.

## Abbreviations

BP: Blood pressure
FHM: Foetal heart monitoring
FHR: Foetal heart rate
LMIC: Low- and middle-income countries
SSA: Sub-Saharan Africa
WHO: World Health Organization

## References

[1] Global Health Observatory data: Maternal and reproductive health. http://www.who.int/gho/maternal_health/en/. 2015. Accessed 3 Aug 2019.

[2] Ollerhead E, Osrin D (2014). Barriers to and incentives for achieving partograph use in obstetric practice in low- and middle-income countries: a systematic review. BMC Pregnancy Childbirth.

[3] Balikuddembe MS, Byamugisha JK, Sekikubo M (2011). Impact of decision – operation interval on pregnancy outcomes among mothers who undergo emergency caesarean section at Mulago hospital. J US-China Med Sci.

[4] Mukasa PK, Kabakyenga J, Senkungu JK, Ngonzi J, Kyalimpa M, Roosmalen VJ (2013). Uterine rupture in a teaching hospital in Mbarara, western Uganda, unmatched case- control study. Reprod Health.

[5] Agarwal K, Agarwal L, Agrawal VK, Agarwal A, Sharma M (2013). Evaluation of paperless partogram as a bedside tool in the management of labor. J Family Med Prim Care.

[6] Kushwah B, Singh AP, Singh S (2013). The Partograph: an essential yet underutilized tool. J Evol Med Dent Sci.

[7] Mandiwa C, Zamawe C (2017). Documentation of the partograph in assessing the progress of labour by health care providers in Malawi’s south-west zone. Reprod Health.

[8] Okokon Ita B., Oku Afiong O., Agan Thomas U., Asibong Udeme E., Essien Ekere J., Monjok Emmanuel (2014). An Evaluation of the Knowledge and Utilization of the Partogragh in Primary, Secondary, and Tertiary Care Settings in Calabar, South-South Nigeria. International Journal of Family Medicine.

[9] World Bank: Uganda makes progress on maternal health but serious challenges remain. In: World Bank news. Available at: http://www.worldbank.org/en/news/feature/2012/10/23/uganda-makes-progress-on-maternal-health-but-serious-challenges-remain. 2012. Accessed 3 Aug 2019.

[10] Essendi H, Johnson FA, Madise N, Matthews Z, Falkingham J, Bahaj AS, James P, Blunden L (2015). Infrastructural challenges to better health in maternity facilities in rural Kenya: community and healthworker perceptions. Reprod Health.

[11] Souza JP, Widmer M, Gülmezoglu AM, Lawrie TA, Adejuyigbe EA, Carroli G, Crowther C, Currie SM, Dowswell T, Hofmeyr J (2014). Maternal and perinatal health research priorities beyond 2015: an international survey and prioritization exercise. Reprod Health.

[12] Balikuddembe MS, Wakholi PK, Tumwesigye NM, Tylleskär T, Ugon A (2018). Midwifery Providers’ preferences for a childbirth monitoring tool in low-income health units in Uganda. Building Continents of Knowledge in Oceans of Data: The Future of Co-Created eHealth.

[13] Dangal G (2006). Preventing prolonged labor by using Partograph. Int J Gynecol Obstet.

[14] American College of Nurses-Midwives (ACNM) (2010). Intermittent auscultation for intrapartum fetal heart rate surveillance. ACNM clinical bulletin number 11, march 2010 (replaces number 9, march 2007). J Midwifery Womens Health.

[15] Neal JL, Lowe NK, Patrick TE, Cabbage LA, Corwin EJ (2010). What is the slowest-yet-normal cervical dilation rate among nulliparous women with spontaneous labor onset?. J Obstet Gynecol Neonatal Nurs.

[16] Sholapurkar S (2010). Intermittent auscultation of fetal heart rate during labour - a widely accepted technique for low risk pregnancies: but are the current national guidelines robust and practical?. J Obstet Gynaecol.

[17] Clark SL, Hamilton EF, Garite TJ, Timmins A, Warrick PA, Smith S (2017). The limits of electronic fetal heart rate monitoring in the prevention of neonatal metabolic acidemia. Am J Obstet Gynecol.

[18] Tandu-Umba NFB, Muamba GK (2015). Using alert and action expected times of delivery in prevention of prolonged labor. Open J Obstet Gynecol.

[19] American College of Obstetricians and Gynecologists (ACOG), Society for Maternal-Fetal Medicine (SMFM) (2014). Safe prevention of the primary cesarean delivery. Obstetric care consensus no. 1. Am J Obstet Gynecol.

[20] World Health Organization (2018). WHO recommendations: intrapartum care for a positive childbirth experience.

[21] Cohen WR, Friedman EA (2015). Misguided guidelines for managing labor. Am J Obstet Gynecol.

[22] Cohen WR, Friedman EA (2015). Perils of the new labor management guidelines. Am J Obstet Gynecol.

[23] Bedwell C, Levin K, Pett C, Lavender DT (2017). A realist review of the partograph: when and how does it work for labour monitoring?. BMC Pregnancy Childbirth..

[24] Oladapo OT, Souza JP, Fawole B, Mugerwa K, Perdoná G, Alves D, Souza H, Reis R, Oliveira-Ciabati L, Maiorano A (2018). Progression of the first stage of spontaneous labour: a prospective cohort study in two sub-Saharan African countries. PLoS Med.

[25] Balikuddembe Michael S, Tumwesigye Nazarius M, Wakholi Peter K, Tylleskär Thorkild (2017). Computerized Childbirth Monitoring Tools for Health Care Providers Managing Labor: A Scoping Review. JMIR Medical Informatics.

[26] International Confederation of Midwives (2014). The future research, education and practice priorities for realizing the potential of the partograph. The 30th triennial 2014.

[27] Hasson F, Keeney S, McKenna H (2000). Research guidelines for the Delphi survey technique. J Adv Nurs.

[28] Hsu C-C, Sandford BA (2007). The Delphi technique: making sense of consensus. Pract Assess Res Eval.

[29] Lenters LM, Wazny K, Webb P, Ahmed T, Bhutta ZA (2013). Treatment of severe and moderate acute malnutrition in low- and middle-income settings: a systematic review, meta-analysis and Delphi process. BMC Public Health.

[30] World health organization: managing complications in pregnancy and childbirth: a guide for midwives and doctors, 2 edn. Geneva: World Health Organization; 2017.

[31] Habibi A, Sarafrazi A, Izadyar S (2014). Delphi technique theoretical framework in qualitative research approach. IJES..

[32] Housseine Natasha, Punt Marieke C., Browne Joyce L., van ‘t Hooft Janneke, Maaløe Nanna, Meguid Tarek, Theron Gerhard B., Franx Arie, Grobbee Diederick E., Visser Gerard H.A., Rijken Marcus J. (2018). Delphi consensus statement on intrapartum fetal monitoring in low-resource settings. International Journal of Gynecology & Obstetrics.

[33] Maaløe N, Housseine N, van Roosmalen J, Bygbjerg IC, Tersbøl BP, Khamis RS, Nielsen BB, Meguid T (2017). Labour management guidelines for a Tanzanian referral hospital: the participatory development process and birth attendants’ perceptions. BMC Pregnancy Childbirth.

[34] Cahill AG, Tuuli MG (2013). Labor in 2013: the new frontier. Am J Obstet Gynecol.

[35] James D (2001). Caesarean section for fetal distress: the 30 minute yardstick is in danger of becoming a rod for our backs. BMJ (Clinical research ed).

[36] Cahill AG, Tuuli MG, Stout MJ, López JD, Macones GA (2018). A prospective cohort study of fetal heart rate monitoring: deceleration area is predictive of fetal acidemia. Am J Obstet Gynecol.

[37] Thomas J, Paranjothy S, James D (2004). National cross sectional survey to determine whether the decision to delivery interval is critical in emergency caesarean section. BMJ..

[38] Parer JT, King T, Flanders S, Fox M, Kilpatrick SJ (2006). Fetal acidemia and electronic fetal heart rate patterns: is there evidence of an association?. J Matern Fetal Neonatal Med.

[39] Ali A, Hummeida M, Elhassan Y, Mnabag W, Ahmed M, Adam G (2016). Concept of defensive medicine and litigation among Sudanese doctors working in obstetrics and gynecology. BMC Med Ethics.

[40] Jünger S, Payne SA, Brine J, Radbruch L, Brearley SG (2017). Guidance on conducting and REporting DElphi studies (CREDES) in palliative care: recommendations based on a methodological systematic review. Palliat Med.

[41] Heelan L (2013). Fetal monitoring: creating a culture of safety with informed choice. J Perinat Educ.

